# Anaesthetic management of cytoreductive surgery followed by hyperthermic intrathoracic chemotherapy perfusion

**DOI:** 10.1186/1749-8090-9-125

**Published:** 2014-07-25

**Authors:** Christoph Kerscher, Michael Ried, Hans-Stefan Hofmann, Bernhard M Graf, York A Zausig

**Affiliations:** 1 Department of Anaesthesiology, University Medical Center Regensburg, Franz-Josef-Strauß-Allee 11, Regensburg, Germany; 2 Department of Cardiothoracic Surgery, University Medical Center Regensburg, Franz-Josef-Strauß-Allee 11, Regensburg 93042, Germany

**Keywords:** Anaesthetic management, Cytoreductive surgery, Hyperthermic intrathoracic chemotherapy perfusion, Thoracic epidural analgesia, One lung ventilation

## Abstract

**Background:**

Macroscopic cytoreductive surgery and hyperthermic intrathoracic chemotherapy perfusion (HITHOC) is a new multimodal approach for selected patients with primary and secondary pleural tumors, which may provide the patient with better local tumor control and increased overall survival rate.

**Methods:**

We present a single-center study including 20 patients undergoing cytoreductive surgery and HITHOC between September 2008 and April 2013 at the University Medical Center Regensburg, Germany. Objective of the study was to describe the perioperative, anaesthetic management with special respect to pain and complication management.

**Results:**

Anaesthesia during this procedure is characterized by increased intrathoracic airway and central venous pressure, hemodynamic alterations and the risk of systemic hypo- and hyperthermia. Securing an adequate intravascular volume is one of the primary goals to prevent decreased cardiac output as well as pulmonary edema. Transfusion of packed red blood cells (PRBC) was necessary in seven of 20 (35%) patients. Only two patients (10%) showed an impairment of coagulation in postoperative laboratory analysis. Perioperative forced diuresis is recommended to prevent postoperative renal insufficiency. Supplementary thoracic epidural analgesia in 13 patients (65%) showed a significant reduction of post-operative pain compared with peroral administration of opioid and non-opioid analgesics.

**Conclusion:**

This article summarizes important experiences of the anaesthesiological and intensive care management in patients undergoing HITHOC.

## Background

In spite of intensive research efforts, there has not yet been any success in establishing a standard treatment regime with curative intention for patients with primary (malignant pleural mesothelioma) and secondary (thymoma Masaoka stage IVa) malignant pleural tumors. Recently a new multimodal treatment approach, including radio-, chemotherapy and macroscopic complete cytoreductive surgery (pleurectomy/decortication or extrapleural pneumonectomy) combined with HITHOC is available, which must be analysed as a potential curative treatment strategy in the upcoming years [1-3]. According to the success of cytoreductive surgery and intraperitoneal hyperthermic perfusion chemotherapy (HIPEC) for peritoneal malignancies [4], results of first studies are promising reaching a decreased recurrence rate and an improved long-term survival [5,6]. Although in the meantime the intra- and postoperative morbidity and mortality depending on patient’s disorders, intraoperative cytostatic concentrations [7] and the degree of surgical resection [8] could be reduced, cytoreductive surgery and HITHOC is a real challenge for the anaesthesiologist with respect to intraoperative management and postoperative pain therapy. Due to this issue being seldom observed and reported in literature so far [9] anaesthetic management is described and discussed in the following report in detail.

## Methods

This study is a retrospective review of 20 patients with primary and secondary malignant pleural tumors who underwent pleurectomy/decortication (P/D: n = 18) or extrapleural pneumonectomy (EPP: n = 2) followed by HITHOC with cisplatin at the Department of Thoracic Surgery of the University Medical Center Regensburg between September 2008 and April 2013. The ethics committee of the University of Regensburg approved the study protocol. An article describing the surgical procedure of this clinical trial has already been published [6]. To protect confidentiality, all data have been anonymised.

### Premedication

The day before surgery, all patients were visited for a detailed history and physical examination by the anaesthesiologist. Particular emphasis was placed on cardiopulmonary risk evaluation according to the recommendations of national and international scientific societies [10,11]. To evaluate the operability, functional lung testing (clinical evaluation, spirometry, blood gas analysis) was performed on all patients. For preoperative anxiolysis patients were treated orally with dikaliumclorazepat 10 – 20 mg (Tranxilium®, Sanofi-Aventis GmbH, Frankfurt am Main, Germany) in the evening and midazolam 3.75 - 7.5 mg (Dormicum®, Hoffmann-La Roche AG, Grenzach-Wyhlen, Germany) in the morning before surgery.

### Induction

On admission to the operating theater, all patients were monitored by electrocardio-gram, pulse oximetry, and invasive arterial blood pressure (radial artery). Before induction of general anaesthesia 13 of 20 patients (65%) additionally received a thoracic epidural catheter in sitting position (mean Th 7/8, range Th 6/7-8/9). After a test dose of 3 ml of ropivacaine 0.75% (Naropin®, AstraZeneca GmbH, Wien, Austria), further 7 ml of ropivacaine 0.75% and sufentanil 10 μg (Sufentanil Curamed®, DeltaSelect GmbH, Dreieich, Germany) was administered in the epidural space. After preoxygenation, general anaesthesia was induced with propofol 2–3 mg•kg^-1^ (Propofol-®Lipuro, B. Braun Medical AG, Sempach, Switzerland), fentanyl 2–4 μg•kg^-1^ (Fentanyl®-Janssen, Janssen Pharmaceutica N. V., Beerse, Belgium) and rocuronium 0.6 mg•kg^1^ (Esmeron®, MSD Merck Sharp & Dohme AG, Luzern, Switzerland), and maintained with inhaled sevoflurane (Sevoflurane®, Baxter Deutschland GmbH, Unterschleißheim, Germany). Intraoperative analgesia was provided with repeated epidural administration of local anaesthetics (ropivacaine 0.75%), or intravenous doses of fentanyl in patients without epidural catheter. For mechanical ventilation with lung isolation, the trachea was intubated with a 39- (respectively 37-) French (Fr) left-sided double-lumen tube, and correct placement was confirmed with fiberoptic bronchoscopy. Additionally central venous pressure (internal jugular vein), oesophageal and buccal body’s core temperature, CO_2_ concentration, airway pressure and urinary output were monitored. Finally, patients were placed in a lateral position on a heating/cooling blanket (Bair Hugger®, Arizant Medical, Inc., Eden Prairie, MN, USA) for maintaining normal body temperature and stabilized with pneumatic boots. All patients received prophylactic antibiotics preoperatively (cefuroxime 1.5 g). Immediately before starting the operation the bronchial or tracheal lumen of the double–lumen tube was clamped and disconnected, and oxygenation with one lung ventilation (OLV) started. The most important goal during OLV is to ensure adequate oxygenation and to prevent hypoxemia as well as acute lung injury (ALI). Therefore, we used protective lung ventilation (PLV) with low tidal volumes (5 ml kg^-1^), limiting plateau pressure and positive end-exspiratory pressure (PEEP) of approximately 5–10 cm H_2_O. Oxygen administration with or without continuous positive airway pressure (CPAP) to the non-ventilated lung was an effective treatment to improve the arterial oxygenation.

### Surgery

In the current study patients underwent a anterolateral thoracotomy through the fifth intercostal space with the aim of an extensive cytoreductive surgery performed with lung-sparing radical P/D or more invasive EPP, when necessary to achieve macroscopic complete tumour resection. Patients with thymoma primarily had radical thymectomy via median sternotomy. In 14 patients (70%) a second access thoracotomy was necessary to complete the surgical cytoreduction in the phrenicocostal recess, or for partial resection of the diaphragm. Extrapleural dissection between the parietal pleura and the chest wall, diaphragm, and mediastinum was continued until the hilar structures were free. After separating the visceral pleura from the lung, wedge resections of infiltrated parts of the lung were performed, if required. Extended resections (partial or complete) of the pericardium, diaphragm, or chest wall were reconstructed with bovine or polytetrafluor-ethylene patches. After completion of the surgical cytoreduction larger air leakages were sutured, chest tubes (24 Fr) were inserted and the unventilated lung expanded.

### Chemotherapy

After wound closure of the thorax a ThermoChem^TM^ HAT-1000 System (Therma-Solutions®, White Bear Lake, MN, USA) was set up to establish a continuous thoracic perfusion. There were two or three inflow catheters placed apically in the anterior and posterior pleural cavity and one or two outflow catheters placed caudally in the anterior and posterior diaphragm sinus. Temperature was measured continuously by two sensors in the inflow and outflow catheters. The priming volume (2–4 L sodium chloride solution or Ringer’s lactate solution) was pumped into the pleural cavity until stabilization of the circulation and homogenous intrathoracic temperature of 42°C were reached. Afterward, cisplatin (100 to 150 mg•m^-2^ body surface area) was administered and perfusion was continued for one hour with approximately 1.2 – 1.5 L•min^-1^ pump flow. In the meantime the application of peep (5–10 mmHg) may increase the lung surface for cisplatin and reduce or prevent pulmonary atelectasis. At the end of the procedure, the perfusion fluid was removed and the tubes were disconnected and left in the pleural cavity as standard thoracic drains with mild suction (20 cm H_2_O) [6].

### Postoperative management

Extubation of the lung directly after surgery in the operation theater was preferred in all cases. Postoperatively all patients were transferred to the cardiothoracic intensive care unit (ICU) and received standard monitoring. For postoperative pain therapy, a continuous epidural infusion with ropivacaine 0.2% and sufentanil 1 μg•ml^-1^ was administered at 7 ml•h^-1^ in patients with epidural catheter. Additional bolus doses of 5 ml ropivacaine 0.2% up to every 45 min for on-demand self-administration were set in the patient-controlled epidural analgesia (PCEA) infusion pump. As a basic pain treatment, all patients received oxycodone/naloxone 10/5 mg (Targin®, Mundipharma GmbH, Limburg (Lahn), Germany) orally every 12 hours and metamizol 1 g (Novamin-sulfon-ratiopharm®, Ratiopharm GmbH, Ulm, Germany) every 6 hours, and for acute treatment morphine 10 mg respectively hydromorphone 1.3 mg (drugstore, University Medical Center Regensburg, Germany) as needed. As a supplement, the administration of ibuprofen 400 mg (Ibuhexal®, Hexal AG, Holzkirchen, Germany), orally, every 8 hours was possible. To optimize pain therapy all patients were visited by an anaesthesiologist from the acute pain service twice a day over a five day period. The sufficiency of the postoperative analgesia was evaluated by the numerical rating scale (NRS), with zero representing no pain and 10 being the worst pain possible. The aim was to achieve a pain intensity of three or less on the NRS.

Patients undergoing cytoreductive surgery and HITHOC are at very high risk of venous thromboembolism. Preoperatively we started thromboembolism prophylaxis with low-molecular-weight heparin (3000 IU of Mono Embolex®, Novartis Pharma GmbH, Nürnberg, Germany). Six hours after surgery treatment with continuous intravenous unfractionated heparin and a target aPTT range of 50–60 seconds was initiated. After removal of the thoracic epidural catheter high dose of low-molecular-weight heparin (8000 IU of Mono Embolex®, Novartis Pharma GmbH, Nürnberg, Germany) was applicated. To improve patient saftey and to reduce the risk of haematoma, we strictly observed the guidelines of the European Society of Anaesthesiology for regional anaesthesia and antithrombotic agents, especially the recommended time intervals between the administration of anticoagulants/platelet aggregation inhibitors, the thoracic epidural blackade and the removal of catheters [12].

During and after HITHOC treatment the safety of the medical staff including the anaesthesiologist is very important. Routes of exposure to the cytotoxic agents include inhalation, ingestion, injection, and skin contact. It is necessary to reduce risks associated with exposure through the proper use of certain specialized medical supplies and equipment. Some essentials contain imperious sterile gowns, unpowdered latex gloves, a respirator mask, a spill kit, protective eyewear, an impenetrable hazardous waste container, appropriate cytotoxic agent labels, and specially marked linen bags. Body fluids and blood from patients undergoing chemotherapy treatment are said to be contaminated for up to 48 hours, starting from the time of administering the dosage. Institutions in which cytotoxic agents are administered should use guidelines to develop policies, procedures, and educational programs to protect medical staff [13].

The present data were collected retrospectively from the patient’s notes, the anaesthesia protocols (Medlinq-Anästhesie®, Medlinq, Hamburg, Germany), digitally acquired ICU files (MetaVision®, Essen-Kettwig, Germany), and the electronic medical records of the acute pain service (Medlinq-Schmerzvisite®, Medlinq, Hamburg, Germany). Statistical analysis was performed by independent-samples *t*-test (SPSS version 18, Dynelytics AG, Zürich, Switzerland). A p value less than 0.05 was considered statistically significant.

## Results

Between September 2008 and April 2013, a total of 20 patients with pleural malignancies were retrospectively enrolled in this study. Patient characteristics are summarized in Table 1. There were 13 male (65%) and 7 female (35%) patients with a mean age of 55 ± 13,6 years. Ten patients (50%) had histologically confirmed malignant pleural mesothelioma. Ten patients (50%) with pleural manifestation of thymoma (Masaoka stage IVa) were included in this trial as well.

**Table 1:** Demographic characteristics of the 20 patients

**Parameters**	**N or mean ± standard deviation (range)**
Age (years)	55 ± 13,6 (25–72)
Sex (male,female)	13 vs. 7
Weight (kg)	76 ± 8,2 (68–93)
Height (cm)	172 ± 13,3 (160–185)
BMI (kg.m^-2^)	26.4 ± 3,1 (20–31)
ASA grade	-
II	6
III	13
IV	1

(BMI: Body-Mass-Index, ASA: American Society of Anaesthesiologists).

**Figure 1:**
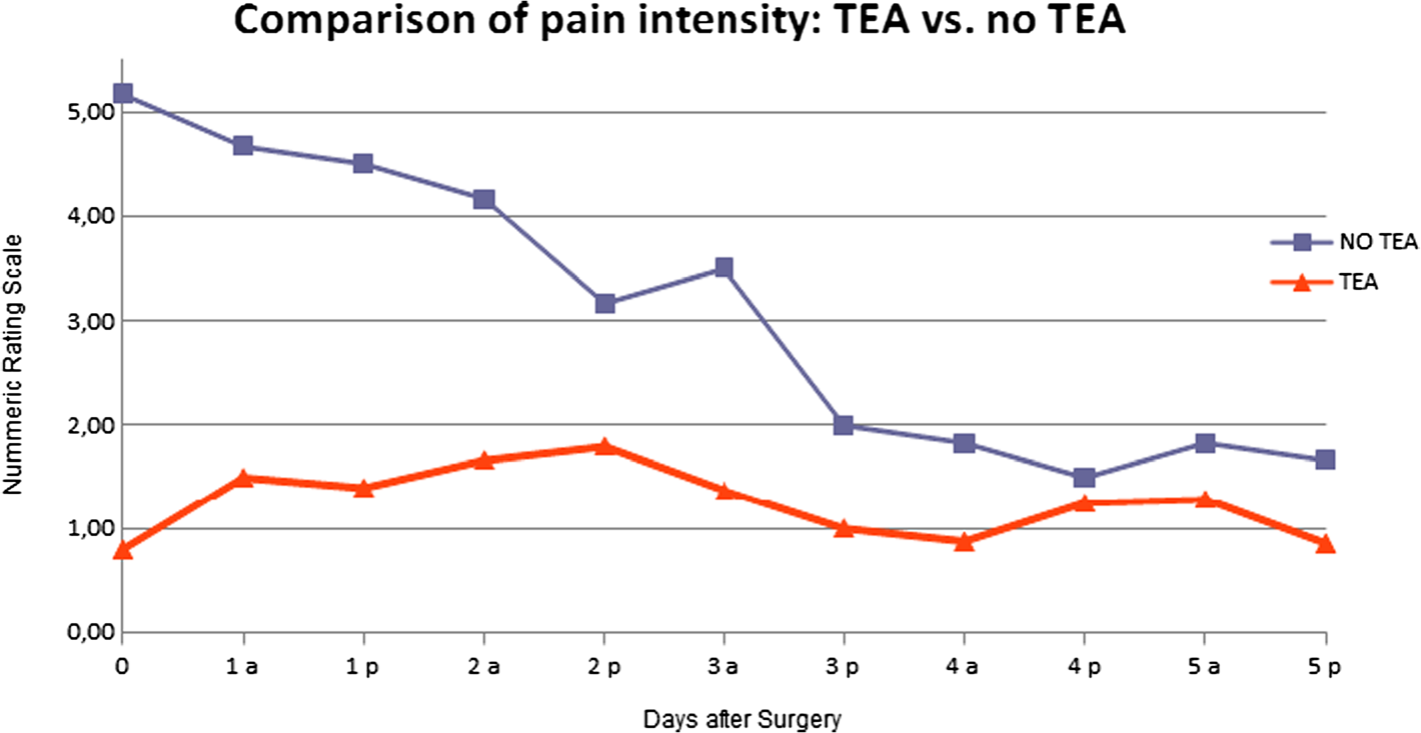
Comparison of pain intensity between the two groups (TEA vs. No TEA) on the first 5 days after surgery at 8 am and 6 pm.

13 of 20 patients (65%) received an epidural catheter before induction of general anaesthesia, four patients refused, two placements were unsuccessful. One epidural catheter was uneventfully removed after a positive blood aspiration. Patients with TEA experienced clinically measurable lower pain intensity in the first 5 days after surgery. Statistically significant lower NRS values were found in the first 60 h after the operation. Mean duration of epidural catheter was 5,2 ± 2 days . Postoperative pain intensity in the two groups is shown in Table 2 and Figure 1.

**Table 2:** Comparison of pain intensity between the two groups (mean ± standard deviation) on the first 5 days after surgery at 8 am and 6 pm

**Day after surgery**	**Time of the day**	**NRS (mean ± SD) TEA**	**NRS (mean ± SD) no TEA**	**p-value**
0	6 pm	0.80 ± 1.47	5.17 ± 1.47	.00*
1	8 am	1.50 ± 1.90	4.67 ± 2.80	.017*
1	6 pm	1.40 ± 1.34	4.50 ± 1.97	.002*
2	8 am	1.67 ± 1.65	4.17 ± 1.32	.009*
2	6 pm	1.80 ± 1.39	3.17 ± 0.75	.046*
3	8 am	1.38 ± 1.18	3.50 ± 0.83	.003*
3	6 pm	1.00 ± 1.22	2.00 ± 1.54	.186
4	8 am	0.88 ± 1.12	1.83 ± 1.60	.212
4	6 pm	1.25 ± 1.48	1.50 ± 1.22	.744
5	8 am	1.29 ± 1.38	1.83 ± 0.98	.435
5	6 pm	0.86 ± 0.89	1.67 ± 0.51	.78

(*p < 0,05 vs. NRS no TEA, NRS: Nummeric Rating Scale, TEA: Thoracic Epidural Analgesia).

During OLV no respiratory complications occurred in any patient. The oxygen saturation and the arterial blood gas measurements did not show any signs of hypoxemia or acidosis induced by hypercapnia.

After application of the priming volume at the beginning of HITHOC ten patients (50%) had a considerable decrease in blood pressure, three patients (15%) additionally developed tachycardia over 100 beats per minute. Simultaneously the mean central venous pressure ranged from 14 to 18 and the peak airway pressure from 26 to 31 mmHg. Meanwhile the mean amount of noradrenaline being infused had to be increased from 0.21 to 0.54 mg•h^-1^. Five patients (25%) additionally received dobutamine at a dose of 10–20 mg•h^-1^, one patient (5%) adrenaline at a dose of 0,2 mg•h^-1^. After strong increase in central venous pressure at the beginning of the intrathoracic perfusion, one patient showed a short episode of asystole (~60 sec.). It was treated successfully by cardiopulmonary resuscitation, application of adrenalin, and reduction of the intrathoracic ringer’s lactate by the perfusion system. There was no evidence of fluid absorption from the pleural space. Extubation was performed 8 hours after surgery without any neurological deficit.

During surgical cytoreduction the average body temperature decreased from 36.6 to 36.3 ± 0,6°C and increased during the HITHOC to 38.4 ± 0,6°C. All patients were passively (with three patients actively) cooled at the beginning of the HITHOC (Bair Hugger®, Arizant Medical, Inc., Eden Prairie, MN, USA).

Severe postoperative complications requiring surgical correction occurred in three patients (15%). Two mesothelioma patients underwent timely reoperation due to a rupture of the diaphragm, one thymoma patient due to a chylothorax. The thymoma patient was readmitted postoperatively to the ICU for 12 days because of respiratory insufficiency and fulminant sepsis. In addition conservative treatment was successful in one patient who experienced subclavian and axillary thrombosis on the ipsilateral side of surgery.

The median duration of the entire surgery including anaesthetisation, intervention and HITHOC was 275 ± 83 minutes. Timely extubation directly after surgery was performed in 15 patients (75%), whereas five patients required mechanical ventilation on ICU - three patients with TEA (23%) and two patients without (28,5%) (p = 0,625). No significant difference resulted in length of postoperative mechanical ventilation between the two groups (406 ± 245 versus 272 ± 60 minutes) (p = 0,144) . The average length of stay on the ICU was 1 ± 0,6 days and the overall median hospital stay was 15 ± 9 days in patients with TEA and 16 ± 10 days in patients without (p = 0,698). One patient died due to a rupture of the diaphragm patch causing pericardial tamponade followed by severe cerebral hypoxia on the second postoperative day after EPP. No further pulmonary complications were observed. There was no other in-hospital mortality or death within 30 days after operation among the other 19 patients in this study.

While in surgery, all patients received a mean volume of 2531 ml crystalloid and 719 ml colloid solutions. Seven patients (35%) with either a hemoglobin concentration below 8 g/dl or clinical transfusion triggers required PRBC. No transfusion of fresh frozen plasma (FFP) or other clotting factors were necessary in any patient. Urine output per kg of body weight was about 2.5 ml/h (Table 3).

**Table 3:** Intraoperative fluid management

**Fluids**	**n (patients)**	**Mean ± standard deviation (range) in ml**
Crystalloids	20	2531 ± 734 (1500–3500)
Colloids	20	719 ± 466 (0–1500)
FFP	0	0
PRBC	7	392 ± 196 (0–750)
Urine	20	877 ± 523 (250–3000)

(FFP: Fresh Frozen Plasma, PRBC: Packed Red Blood Cells).

Postoperative blood results on ICU (Table 4) suggested that there were only two patients (10%) with the presence of abnormal blood clotting. The mean hemoglobin values were over 8 g/dl in all 20 patients. One patient developed an acute renal insufficiency with a creatinine concentration of 2.7 mg/dl three days after surgery. Dialysis was not necessary in this case. All other patients did not show any significant alterations compared to control levels.

**Table 4:** Pre- and postoperative laboratory parameters: mean ± standard deviation, (range)

**Variables**	**Preoperative parameters**	**Postoperative parameters (Day 0)**	**Postoperative parameters (Day 3)**
Hemoglobin (g/dl)	12.4 ± 1.93, (8.4 – 15.3)	9.92 ± 1.16, (8.6 – 11.9)	
Hematocrit (%)	36.4 ± 5.56, (22.5 – 43.7)	28.9 ± 3.36, (24.6 – 33.6)	
Creatinine (mg/dl)	0.85 ± 0.18, (0.66 – 1.20)	0.84 ± 0.17, (0.45 – 1.25)	0.97 ± 0.49, (0.44 - 2.7)
Prolonged time (%)	105 ± 8.57, (92 – 127)	80.8 ± 11.12, (46–92)	
PTT (sec)	30.7 ± 3.53, (25.8 – 38)	32.7 ± 6.94, (24.1 – 52.0)	
Thrombocytes (/nl)	347 ± 135.5, (178 – 699)	274 ± 97.5, (149–511)	

## Discussion

The indication for cytoreductive surgery in combination with HITHOC was restricted for the treatment of malignant pleural mesothelioma and advanced thymoma with pleural spread for a long time. During the last years a few authors showed that this multimodal approach could be a feasible treatment option for patients with pleural dissemination of pseudomyxoma peritonei, peritoneal mesothelioma or epithelian ovarian cancer, as well [9,14,15]. Hence the indications for this treatment are probably growing, and not only at specialized medical centers [16]. Due to the complex pathophysiological changes accompanying P/D or EPP and HITHOC, careful scheduling and implementation of the anaesthesia is of utmost importance for patient safety.

At the beginning of chemotherapy perfusion, thoracic filling with hyperthermic ringer’s lactate causes an increase in intrathoracic pressure. Depending on the volume of solution, a mediastinal shift to the ventilated lung resulting in an increase in airway pressure and a reduction of the functional residual capacity can be observed [17-19]. Therefore obstruction of the inferior and superior vena cava decreasing venous return to the heart, and direct cardiac compression can lead to a fall in cardiac output. In our understanding these pathophysiological alterations resulted in a reduction of the arterial blood pressure and the tissue perfusion, followed by a considerable rise of catecholamine levels in this trial. In addition, compression-induced increase of the myocardial wall tension and the heart rate can also cause an acute rise of the oxygen demand, and in combination with reduced coronary perfusion pressure exacerbate existing cardiac disease in patients with coronary artery disease.

Perioperative fluid management is an important cornerstone of hemodynamic stability in patients undergoing cytoreductive surgery followed by HITHOC and a challenge for each anaesthesiologist. Recent studies and reviews have repeatedly demonstrated a strong correlation between liberal fluid administration and the incidence of postoperative lung edema or ALI. Especially in patients undergoing lung surgery, excessive volume infusion can contribute to respiratory failure with increased postoperative mortality [20-22]. On the other hand, an adequate perioperative fluid therapy is important to reduce the risk of chemotherapy-related postoperative renal insufficiency. Therefore we recommend a perioperative forced diuresis (>100 ml/h), which will be continued for 3 days after surgery. Furthermore the aim of perioperative fluid therapy also is to replace fluid losses, to maintain adequate intravascular volume in the cytoreductive phase, and to ensure a sufficient preload before starting the chemotherapy perfusion, with the above-mentioned pathophysiological changes. For this the implementation of hemodynamic monitoring (pulmonary artery catheter, pulse contour cardiac output) could be an adjuvant tool. In addition the application of the priming volume must be started under accurate control of hemodynamic and ventilatory parameters, and from close consultation with the surgeons. For compensation of hemodynamic disorders, vasopressor and inotropic drugs have to be prepared before starting surgery. Three principles could help to prevent complication seen in our patients: an adequate fluid therapy, a careful filling of the thoracic side, and an early use of catecholamines.

Maintaining patient normothermia during the whole procedure should be another goal of anaesthetic management. Therefore the patient’s body temperature was continuously evaluated buccally and esophageally. During the first phase of anaesthesia (induction, cytoreductive surgery) patients have to be protected from loss of body heat. By using warming blankets and heated fluid infusions, excessive hypothermia could be prevented in our patients. In only one case a temperature was measured under 35.5°C. Recent studies showed that perioperative hypothermia occurs in more than 70% of patients in the perioperative phase and can lead to several complications: increase in blood loss during surgery by impairing platelet function and clotting factor enzyme function, increase in the incidence of surgical wound infection induced by peripheral vasoconstriction with a significant reduction of subcutaneous oxygen tension and an impairment in immune function, increase in heart rate and oxygen demand in the presence of shivering [23-25]. Additionally Frank et al. reported a higher prevalence of myocardial ischemia and ventricular tachycardia in patients with perioperative hypothermia [26]. Otherwise during HITHOC temperature in the pleural space rises up to about 42°C with the risk of systemic hyperthermia. As we know from patients undergoing cytoreductive surgery and HIPEC, elevated body temperatures can increase metabolic rates and oxygen demand followed by tachycardia, higher end-tidal CO_2_ concentration and a metabolic acidosis [27,28,17,18]. The development of myocardial ischemia is a possible complication especially for patients with existing coronary artery disease. Hyperthermia can also lead to pulmonary edema and ventilator-induced acute lung injury [29,30]. In addition it may contribute to development of neurocognitive dysfunction, which is often described during the rewarming process after cardiopulmonary bypass for cardiac surgery [31]. Animal experiments showed that hyperthermia with temperatures over 43°C can lead to neurological and electrophysiological alterations in peripheral nerves like such as phrenic, vagus and recurrent laryngeal nerves [9,32]. Thereby hyperthermia can possibly contribute to a modified apperception of pain. To avoid systemic hyperthermia in this phase, we used cooled infusions and cooling blankets. In spite of extensive cooling nine of our patients developed core body temperatures above 38.0°C, three patients even above 38.5°C. There were no hyperthermia-induced complications in the current cohort of patients. Regional intracavitary chemotherapiy perfusion has the advantage of an improved distribution of the drug in the pleural space and a higher local concentration of the chemotherapeutic agent in contrast to systemic chemotherapy. Additionally, HITHOC is known to enhance the cytotoxicity of some chemotherapeutics, to increase the penetration depth, and to produce synergistic antineoplastic effects. Although the systemic toxicity could be reduced, significant blood concentrations of the cytostatic agents were measured [33-35]. While toxicities of cisplatin include ototoxicity, gastrotoxicity, myelosuppression and allergic reactions, the main dose-limiting side effect is nephrotoxicity. To prevent this cytostatica-induced tubular nephrotoxicity, the aim of the anaesthetist should be a forced perioperative hydration and diuresis. Therefore a urine flow greater than 0.5 ml kg^-1^ h^-1^ should be achieved, although no randomized studies can acknowledge this approach [36].

The retrospective analysis of our data shows that about one third of the patients (35%) received a PRBC transfusion or cell saver blood with subsequent irradiation (50 Gy). Comparable transfusion rates (34.6%) were reported for patients undergoing cytoreductive surgery and HIPEC [18]. Furthermore, due to an impairment of coagulation, about 45% of HIPEC patients received a transfusion of fresh frozen plasma as well. Otherwise laboratory analysis of our patients demonstrated no such disturbance of coagulation and so the transfusion of fresh frozen plasma or thrombocytes was not necessary. The reason for coagulopathy may be a large volume shift and a significant protein loss depending on the degree of decortication and the volume of fluid resuscitation. In our collective we also could not find any direct effect of the HITHOC itself [18,37]. Due to a large raw peritoneal surface area, impairment of coagulation is more often associated with cytoreductive surgery of the abdominal than of the thoracic cavity.

Thoracotomy induces severe postoperative pain and impairment of pulmonary function. Therefore the TEA is commonly considered the “gold standard” [38]. In our trial, TEA was superior to peroral opioid and non-opioid analgesics. Significantly lower values of NRS at rest were found in the first 60 h after surgery in the epidural group. No significant difference resulted in duration of postoperative mechanical ventilation, incidence of postoperative pulmonary complications or median length of hospital stay between the two groups. Postoperative lung function testing, which would probably show differences between the two groups were, not part of standard postoperative management. However, multiple former studies have also demonstrated a superior functional residual capacity, vital capacity and forced exspiratory volume after the administration of epidural analgesia, and in addition, an improvement of the bowel motility and function, the postoperative vigilance and the balance of myocardial oxygen supply and demand [39-41].

## Conclusion

Thoracic cytoreductive surgery and intraoperative HITHOC is an innovative, multimodal approach with a potentially curative intention for patients with primary and secondary malignant pleural tumors. In this article we reviewed the pathophysio-logical alterations and emphasized anaesthetic management strategies to avoid or minimize complications and to improve peri- and postoperative care of patients.

### Consent statement

Written informed consent was obtained from the patients for publication of this case report and any accompanying images. A copy of the written consent is available for review by the Editor-in-Chief of this journal.

## Abbreviations

ALI: Acute lung injury; CPAP: Continuous positive airway pressure; EPP: Extrapleural pneumonectomy; FFP: Fresh frozen plasma; Fr: French; Gy: Gray; HITHOC: Hyperthermic intrathoracic chemotherapy perfusion; HIPEC: Intraperitoneal hyperthermic perfusion chemotherapy; ICU: Intensive care unit; NRS: Numerical rating scale; OLV: One lung ventilatíon; PCEA: Patient-controlled epidural analgesia; Peep: Positive end-exspiratory pressure; PLV: Protective lunge ventilation; P/D: Pleurectomy/Decortication; PRBC: Packed red blood cells; TEA: Thoracic epidural analgesia.

## Competing interest

The authors declare that they have no competing interests.

## Authors’ contributions

CK and YZ conceived of the study, CK collected the present data, performed the statistical analysis and drafted the manuscript, MR, HSH and BG participated in its design and coordination and helped to draft the manuscript. All authors read and approved the final manuscript.
